# A survey on nasoalveolar moulding treatment practices at cleft centres across India

**DOI:** 10.3389/fsurg.2025.1526364

**Published:** 2025-03-05

**Authors:** Badri Thiruvenkatachari, Thailavathy Vaidhyalingam, Subhiksha Chakkaravarthi, Manoj Prathap, Karthika Nambiar

**Affiliations:** Sree Balaji Dental and College and Hospital, Bharath Institute of Higher Education and Research, Chennai, India

**Keywords:** cleft, nasoalveolar molding, cleft lip and palate (CLP), NAM, survey, cross sectional study

## Abstract

**Background:**

The purpose of this study is to assess the current protocols followed in the practice of NAM treatment for patients with cleft lip and palate across different comprehensive centres in India.

**Design:**

Cross sectional questionnaire based study.

**Method:**

Comprehensive cleft teams across India were invited to participate in this survey. The questionnaire was developed over four stages, with a panel of eight members. The developed questionnaire consisted of 29 questions that included demographic details, decision-making process, treatment protocols, experiences with treating patients, difficulties and complications encountered during treatment. The results were reported descriptively in percentages.

**Results:**

Of the 46 teams, 39 teams (85%) reported offering NAM before lip surgery, while 15% (*n* = 7) teams did not offer NAM. Of these 39 teams, almost half (49%, *n* = 19) of the teams were providing NAM to less than 20% of their patients, 28% (*n* = 11) of the teams were providing treatment to more than 50%, and the remaining respondents reported (23%, *n* = 9) providing NAM to 21%–50% of their patients. There is a consensus that NAM is beneficial for both unilateral and bilateral clefts, with the Grayson's method as the most commonly used technique. While there is general agreement on their effectiveness, 15% of participants were unsure. The most frequently reported side effects were cheek and mucosal irritation. Despite these issues, all respondents would recommend NAM treatment.

**Conclusion:**

The survey demonstrated a strong consensus among centers regarding most aspects of NAM treatment. Notably, all respondents expressed their willingness to recommend NAM to their friends and family.

## Introduction

Cleft lip and palate is one of the most common congenital malformations with approximately 28,000–33,000 children are born with cleft lip and palate in India every year (1). Patients with cleft lip and palate usually face a multitude of problems which necessitates a multidisciplinary approach involving various medical and dental specialties that extends from birth to adulthood. One of the first steps in cleft care is the functional and aesthetic correction of cleft lip, which continues to be a major challenge for the healthcare team. More importantly, the challenge is to retain the functional/aesthetic correction achieved in the first year. Nasoalveolar moulding (NAM) is an adjunctive procedure carried out before lip surgery that involves the active movement of the maxillary fragments with alveolar plates to approximate the cleft segments and to improve the nasal symmetry. There are claims that NAM followed by primary surgeries can reduce the amount of relapse and thereby, reduce the need for revision surgeries (2–4). However, the use of NAM can be an intensive and time-consuming process for both the practitioners and caregivers. The treatment is started as early as five days after birth and involves weekly appointments, daily replacement of surgical tapes, appliance replacement for growth adjustments, all adding up to the increased burden of care. Additionally, there are financial and psychological challenges faced by caregivers of patients undergoing NAM treatment (5–7).

Previous studies have reported a huge variation in the treatment availability and treatment protocol for NAM between centres globally. For example, the Americleft and the Eurocleft studies reported that 65% and 37% of cleft centres provide NAM treatment routinely for their patients with cleft lip and palate (8, 9). Unfortunately, there is no data from Indian centers on access or the NAM protocol followed. Hence, this survey aims to assess the decision-making and treatment protocol for NAM treatment among various clinicians treating patients with cleft across India.

## Methodology

This questionnaire-based cross-sectional study was conducted on a convenience based sample of cleft teams across India. The study protocol was approved by the Institutional Ethics Committee. The survey was conducted from March 2023 to November 2023.

### Questionnaire development

The development of the NAM questionnaire comprised four key stages. Initially, the study's aim was delineated. Subsequently, an exhaustive evaluation of previous systematic reviews on NAM was conducted, culminating in the formulation of the questionnaire draft with input from an expert panel. Notably, existing research shows the requirement of a panel comprising 2–20 experts (10); therefore, our expert panel comprised four cleft orthodontic and two cleft surgical members. During the next phase, the expert panel engaged in probing discussions, addressing queries such as “*thoughts on the effectiveness of NAM*”, and were also presented with a series of open-ended inquiries to stimulate ideation. Subsequent rounds of expert meetings saw the generation of new questions derived from analyses of prior discussions, with concerted efforts made to attain consensus. Following the third round, consensus was successfully achieved, enabling the finalization of the questionnaire by the expert panel.

The developed questionnaire was assessed for content validity (11). An expert panel of five members (four cleft orthodontists and one cleft surgeon) evaluated the questionnaire for content validity. The item level (I-CVI) and scale level (S-CVI/Ave) content validity was evaluated along with scale level content validity index based on the universal agreement method (S-CVI/UA). The results showed 27 of the 30 questions had content validity of 1, two had a CVI of 0.8 and one had 0.4. The S-CVI was 0.94 and the S-CVI/UA was 0.86.

For ease of reporting, the questionnaire was segregated into four domains, (i) seven questions on the demographic details of the team, (ii) two questions on the factors affecting the decision on providing NAM treatment, (iii) 19 questions on their experiences on treating patients with NAM and (iv) two question on the difficulties and complications encountered with NAM.

The survey was created using the SurveyMonkey platform and included participant consent along with an introduction detailing the project's information and objectives. Upon calculating the sample size needed for a 95% confidence interval and a 5% error margin, it was determined that 68 cleft teams across India would need to be surveyed. However, as we decided to include only comprehensive cleft teams (defined as providing surgical, orthodontic, and speech support) across India, we were limited with the number of centres. The questionnaire was emailed to all identified comprehensive cleft centre teams, allowing participants a two-week window to complete it. Telephone reminders were sent at two-week intervals. Teams failing to respond after three calls were considered dropouts. Additionally, we targeted clinicians attending Indian Cleft Conference held in xx in April 2023. Clinicians, including both orthodontists and surgeons, collaborated to answer survey questions collectively. Each center returned one questionnaire for analysis.

### Data analysis

The response from SurveyMonkey was exported to excel sheet directly. The data was then organised for analysis and this was cross checked for errors by the second investigator.

Descriptive statistics was performed and data reported as mean and percentages. Two reviewers independently and in duplicate segregated open ended questions and the answers were then reported descriptively.

## Results

### Demographic details

A total of 46 cleft teams returned the completed questionnaires. The demographic data showed an equal distribution of participants by gender, with 23 males and 23 females. Nearly half of the respondents (46%, *n* = 21) were aged between 36 and 45 years, 20% (*n* = 9) were aged between 46 and 55 years, and 28% (*n* = 13) were aged between 25 and 35 years, while only one respondent was over 55 years old. Among the 46 respondents, 65% (*n* = 30) were orthodontists, 26% (*n* = 12) were oral surgeons, and 9% (*n* = 4) were plastic surgeons (Table 1).

**Table 1:** Demographics, surgical and NAM treatment caseload and reasons for not offering NAM treatment.

	*n* (%)
Work place (*n* = 38) skipped 1	
Public/Govt. hospital	9 (20)
Private practice/hospital	29 (64.44)
Faculty in an institution	4 (8.89)
Other (please specify)	3 (6.67)
At what age of the baby do you prefer doing lip surgery at your centre (*n* = 45; skipped = 1)	
<3 months	6 (13.33)
3–6 months	26 (57.78)
6–8 months	12 (26.67)
9–12 months	1 (2.22)
>12 month	0
Do you provide NASOALVEOLAR MOLDING before lip surgery of CLP new-borns in your centre	
Yes	39 (84.78)
No	7 (15.22)
Percentage of CLP cases undergone NAM in the last year	
0%–20%	19 (48.72)
21%–50%	9 (23.08)
More than 50%	11 (28.21
Reasons for not offering NAM	
My unit lacks the workforce	5 (29.41)
None of the above	7 (41.18)
Other (please specify)	6 (35.29)
We do not have funding for NAM treatment	2 (11.76)
I don't think it is beneficial in the long run	1 (5.88)
I don't think it helps	0
I have no knowledge on NAM	0
There is no evidence for NAM treatment	0

Nearly two-thirds of the participants (64%, *n* = 29) were working privately, while nine (20%) were working in a secondary care public hospital, and four teams (9%) were working in tertiary care attached to an educational institution (Table 1). When asked if they were working with the mentioned Non-Government charity organizations (NGOs), a significant proportion of the respondents reported being affiliated with Smile Train (75%, *n* = 33), while one team was working with Akila Bharatha Mahila Seva Samaja (ABMSS) (2%, *n* = 1), and two teams with Mission Smile (4%, *n* = 2).

### Experience: (*N* = 46)

Nearly two-thirds (63%, *n* = 29) of the teams were providing cleft care for less than 10 years, while one-third (32%, *n* = 15) had 11–20 years of experience in cleft care, and two teams (4%) had more than 20 years of experience (Table 1).

The majority of the teams (58%, *n* = 26) preferred lip surgery to be performed between 3 and 6 months of age, 27% (*n* = 12) preferred it between 6 and 8 months, and 13% (*n* = 6) preferred lip surgery to be performed before 3 months of age, while one team (2%) preferred performing lip surgery between 9 and 12 months of age. None of the teams waited over 12 months to perform lip surgery (Table 1).

Of the 46 teams contacted, 39 teams (85%) reported that they offered NAM before lip surgery, while 15% (*n* = 7) did not offer NAM (Table 1).

### Reasons for not offering

The major reason cited for not using NAM (29%, *n* = 5) was a lack of workforce. Four respondents (33%) had no specific reason for not using NAM. Two centers (17%) specified insufficient funding for providing NAM treatment, while one center considered NAM not beneficial in the long run. Additionally, one team cited poor compliance from patients reporting from remote locations as a reason for not adopting NAM (Table 1).

### Timing of NAM

Four-fifths of the respondents (79%, *n* = 31) preferred starting NAM treatment within the first 3 weeks, while six teams (15%) preferred to start between 3 weeks and 2 months, and two teams (5%) preferred starting between 2 and 4 months. None of the teams preferred initiating NAM treatment after four months of age.

### Percentage of NAM cases

Of these 39 teams, nearly half (49%, *n* = 19) provided NAM to less than 20% of their patients. Additionally, 28% (*n* = 11) of the teams provided treatment to more than 50% of their patients, and 23% (*n* = 9) reported providing NAM to 21%-50% of their patients (Table 1).

### Training for NAM treatment: (*N* = 39)

Almost two-thirds of the respondents 61% (24) had participated in the NAM training programs while the rest 38% (15) had not had any formal training on NAM (Table 2).

**Table 2:** NAM training, technique and preference on treating clinician.

	Responses *n* (%)
Have you previously attended any training for NAM (workshop/CPD courses etc.)	
Yes	24 (61.54)
No	15 (38.46)
What NAM technique do you follow?	
Grayson	24 (61.52)
Figueroa	6 (15.38)
Liou	3 (7.69)
Taylor	0
I don't know	3 (7.69)
Others	6 (15.38)
Who performs NAM in your centre (*N* = 38; skipped = 1	
Prosthodontist	0
Orthodontist	36 (94.74)
Paediatric dentist	2 (5.26)
General dentist	0
Which specialist do you think should manage NAM in patients with cleft? *N* = 39	
General surgeons	0
General nurses	0
Orthodontists	37 (94.87)
Prosthodontist	1 (2.56)
Paediatric dentist	1 (2.56)

### NAM technique

More than half of the respondents showed a preference for Grayson's technique (61%, *n* = 24), while 15% (*n* = 6) were practicing Figueroa's technique. Three respondents (8%) used Liou's technique for NAM treatment. Additionally, three respondents selected the “others” category: one specified using a passive plate, another used a combination of techniques, and the third used Figueroa, Grayson, and their own modified technique (Table 2).

### Specialty involved in managing NAM

In 95% (*n* = 36) of the cleft teams, orthodontists were responsible for performing the NAM procedure. In contrast, 5% (*n* = 2) of the centers had pediatric dentists managing NAM patients (Table 2). When asked about the preferred specialist for managing NAM in patients with cleft, 99% (*n* = 37) of providers recommended referral to orthodontists, while one provider (3%) suggested a prosthodontist and another (3%) preferred a pediatric dentist (Table 2).

Regarding the fabrication of NAM appliances, 62% (*n* = 20) of the centers had the appliance fabricated by the clinician, 31% (*n* = 12) employed technicians for fabrication, and 8% (*n* = 3) reported that it was fabricated by “others”, with no additional details provided (Table 2).

### Unilateral vs. bilateral cleft

Two-thirds of respondents (69%, *n* = 27) agreed that NAM is beneficial for both unilateral and bilateral clefts. In contrast, 26% (*n* = 10) believed that NAM is most beneficial for bilateral clefts, while 5% (*n* = 2) felt that it is most advantageous for patients with unilateral cleft lip and palate (Table 3).

**Table 3:** Indications and recall for NAM.

	*n* (%)
In which type of clefts, presurgical orthopedics (NAM) would be most beneficial? *n* = 39	
Unilateral	2 (5.13)
Bilateral	10 (25.64)
Both	27 (69.23)
How frequent do you recall patients undergoing NAM procedure after the first insertion? *n* = 39	
Every day	1 (2.56)
Every week	1 (43.59)
Once every two weeks	16 (41.03)
Once a month	5 (12.82)

### Frequency of recall visits

Regarding the frequency of recall visits following the initial appointment, 44% (*n* = 17) of respondents reported recalling patients once every week, 41% (*n* = 16) recalled patients every two weeks, and 13% (*n* = 5) recalled every four weeks. Additionally, one team (3%) indicated that they called patients daily (Table 3).

### Effectiveness of NAM

When asked about the usefulness of NAM for patients with cleft, 82% (*n* = 37) of the teams considered NAM beneficial for both unilateral and bilateral clefts, while 18% (*n* = 8) believed it was more useful for bilateral cleft cases (Table 4).

**Table 4:** Effectiveness of NAM.

	Closed-ended questions	Options	*n* (%)
1	Do you think NAM improves the baby's feeding ability?	Yes	33 (84.62)
		No	0
		Not sure	6 (15.38)
2	Do you think NAM improves the baby's facial appearance?	Yes	39 (100)
		No	0
		Not sure	0
3	Do you think NAM influences the surgical repair of cleft lip?	Yes	38 (97.44)
		No	0
		Not sure	1 (2.56)
4	Do you think NAM reduces the alveolar gap?	Yes	39 (100)
		No	0
		Not sure	1 (3.03)
5	Do you think that the NAM treatment resulted in change in shape or size of the baby's nose?	Yes	38 (97.44)
		No	0
		Not sure	1 (2.56)
6	Do you think NAM helps in columellar lengthening?	Yes	35 (89.74)
		No	0
		Not sure	4 (10.26)

The effectiveness of NAM was assessed through six questions. All teams agreed that NAM improves the baby's facial appearance and reduces the alveolar gap. The majority of teams concurred that NAM results in a change in the shape or size of the baby's nose (97%, *n* = 38), influences the surgical repair of the cleft by reducing the alveolar gap (97%, *n* = 38), and aids in columellar lengthening (89%, *n* = 35). However, there was a slight decrease in agreement regarding the improvement in the baby's feeding ability (84%, *n* = 33) (Table 4).

### Challenges faced in managing NAM

When asked about the difficulty of performing the NAM procedure, most respondents (68%, *n* = 26) reported no difficulty, while 18% (*n* = 7) found the procedure challenging. The remaining 13% (*n* = 5) were unsure (Table 5).

**Table 5:** Difficulty rating and complications with NAM.

	*n* (%)
Do you find NAM procedure difficult? *n* = 38	
Yes	7 (18.42)
No	26 (68.42)
Not sure	5 (13.16)
Side effects	
Cheek skin irritation due to tapes	28 (80)
Mucosal irritation in the mouth	16 (45.71)
Tape displacement	15 (42.86)
Lip irritation due to tapes	12 (34.29)
Impingement on nasal epithelium	6 (17.14)
Vomiting	5 (14.29)
Difficulty in feeding	3 (8.57)
Tissue fungal infections	3 (8.57)
Bleeding (intraoral or extraoral)	3 (8.57)
Other (please specify)	4 (11.43)
Sleeplessness	0

Regarding reported problems with NAM treatment at their centers, respondents could select multiple issues. The most commonly reported problem was cheek skin irritation due to tapes (80%, *n* = 28). Other frequent issues included mucosal irritation in the mouth (46%, *n* = 16), tape displacement (43%, *n* = 15), and lip irritation from the tapes (45%, *n* = 13). Additionally, some respondents reported impingement on the nasal epithelium (17%, *n* = 6), while fewer reported vomiting (14%, *n* = 5), difficulty in feeding (8.57%, *n* = 3), tissue fungal infections (9%, *n* = 3), and bleeding (intraoral and extraoral) (9%, *n* = 3). One participant noted problems with fitting issues that required denture adhesives and excessive use of tapes, which made parents reluctant and anxious. Two other participants highlighted issues with patient compliance and difficulties with appliance remodeling (Table 5).

When asked if they would recommend NAM treatment for their own child or a relative with cleft lip and palate, all respondents (100%, *n* = 39) agreed that they would (Table 6).

**Table 6:** Recommending NAM for family and friends.

	*n* (%)
Would you recommend NAM to your child, family or friends? *n* = 38	
Yes	39 (100)
No	0

## Discussion

The present survey shows that majority of the comprehensive cleft centres provide NAM (85%) treatment although almost half of the centres reported to routinely provide this for less than 20% of their reporting patients. Almost everyone agreed that orthodontist should be the one providing treatment, and NAM is beneficial for both unilateral and bilateral cases. Cheek and mucosal irritation seemed to be the most common side effect. They all would recommend this treatment to their family and friends.

### Teams offering NAM

In a 2012 telephone survey regarding NAM practices in the USA, 37% of centers were reported to offer NAM treatment (8). However, a more recent survey reported 86% of the centres in the USA providing NAM treatment (12). Although factors such as caregiver travel distance and financial situation can significantly impact access to care, there has been a substantial increase in the number of centers providing treatment and the percentage of patients receiving care. The results of the present study are similar to the recent US survey with 85% of centres providing NAM treatment. However, the survey was conducted in comprehensive care centers in India, and thus may not accurately reflect the situation of all cleft centers across the country. In a 2010 Indian survey on the management of cleft lip and palate (CLP), out of the 112 centers that participated, 19 (16.96%) reported providing presurgical orthopedic treatment (13). A similar trend to that observed in the USA is seen in India, with a 68% increase in the number of centers offering presurgical orthopedic treatment over the past decade.

### Type of appliance

In a recent study in the USA, of the 112 centers surveyed, 68% were using NAM appliances, while the remaining centers were providing lip taping, Dynacleft, Latham appliances, and passive plates (12). The present study did not identify any centers providing presurgical orthopedics other than NAM. Although the nasoalveolar molding (NAM) appliance appears to be the most popular type of presurgical device, more invasive appliances like the Latham appliances are still in use in the USA. This may be due to the lack of long-term evidence supporting any specific presurgical orthopedic appliance. These findings highlight the need for further high-quality, long-term studies in this area.

### Reasons for not offering NAM

Previous surveys on NAM have cited the distance patients need to travel and financial reasons as the main barriers to providing NAM treatment (7, 12). Other common reasons include a lack of clinicians with the necessary skills, no evidence of benefit compared to the high burden of care, and patient noncompliance or refusal. The present study showed similar results, with the majority of centers reporting a lack of skilled clinicians. Interestingly, in this study, none of the center clinicians cited a lack of evidence as a reason for not providing NAM. Not surprisingly, two respondents reported funding as a reason for not providing NAM. This situation is unique to India and other low and lower-middle-income countries, as they are primarily funded by non-government charity organizations (NGO), with limited funding for centers not supported by NGOs (14). Interestingly, there were no reports on the financial implications for patients as a reason for not providing treatment. It is important to note that more clinical trials are needed on the long-term effectiveness of NAM and, more importantly, the health economics of NAM treatment.

The burden on patients has been discussed in previous studies (15, 16). A recent study highlighted that caregivers experienced positive psychosocial benefits, including reduced anxiety and depression, compared to those who received usual care (17). The study reported a rapid decline in anxiety and depression and improved coping among caregivers. Although the present study did not evaluate the burden on caregivers, none of the study centers reported patient burden as a reason for justifying NAM treatment.

When financial situation and location were taken into consideration, a previous study in the United States reported that neither was a reason for not adopting NAM (7). They also noted that age at presentation was an important factor for not providing NAM treatment. The results are similar to the present study, with only one center reporting travel distance to the unit as a reason for poor compliance and two centers reporting late presentation as a reason for poor compliance.

### Percentage of cleft cases undergoing NAM

In a 2012 survey on NAM, 90% of participants used plaster strapping before surgical repair, and only 8.3% routinely practiced NAM (18). Interestingly, 11.7% provided feeding plates for their patients. The results of this study differ markedly from the present study for two reasons: the earlier study was conducted more than a decade ago, and the NAM technique has gained significant popularity since then. Additionally, the previous study included only 6 orthodontists out of 60 respondents, whereas the present study had an orthodontist at every included center.

### NAM timing

The timing of NAM treatment is widely accepted as an important indicator of success. In a previous study, the authors showed that starting NAM within the first four weeks after birth produced the best results (19). A similar study reported that age at presentation was the only significant variable in the success of NAM treatment (7). Matsuo recognized that the cartilage in newborns is soft and lacks elasticity. He believed that the high level of estrogen at birth correlates with increased hyaluronic acid, which inhibits the linking of the cartilage intercellular matrix. Therefore, undertaking nasal cartilage molding as early as possible is advantageous to achieve more long-lasting molding of the relatively plastic immature cartilage and avoid the elastic rebound that would result from older, more mature, and less plastic cartilage. The present study shows a similar trend, with nearly 80% of respondents reporting that they prefer to start treatment within the first three weeks.

### Unilateral vs. bilateral cases

Although NAM treatment is prescribed for both unilateral and bilateral cases, it is widely accepted that NAM is more effective in bilateral cases. Understandably, the premaxillary segment in bilateral cases pose a much bigger challenge for the surgeons than the wide unilateral clefts. The results from a previous study showed that NAM produced more nasal symmetry and greater increases in both columnar height and width in bilateral patients compared to patiens with unilateral cleft lip and palate (20). The results from the present study showed that more centres favoured bilateral cases than unilateral.

Although Nasoalveolar Molding (NAM) treatment is prescribed for both unilateral and bilateral cases (3), its effectiveness is notably higher in bilateral cases (16). The premaxillary segment in bilateral cases poses a significantly greater surgical challenge compared to wide unilateral clefts. Previous research indicates that NAM results in superior nasal symmetry and greater increases in both columnar height and width among bilateral patients compared to those with unilateral cleft lip and palate (20). Additionally, findings from our study reveal a prevailing preference among treatment centers for managing bilateral cases over unilateral ones.

### Challenges with NAM

The primary goal of NAM treatment is to reduce cleft severity and shape the nose prior to lip surgery. Numerous studies have documented high success rates in narrowing the cleft width, although evidence regarding improvements in nasal aesthetics remains limited (21–23). Despite its efficacy, NAM is associated with several side effects that necessitate thorough discussion with parents or caretakers before initiating treatment. Reported side effects include cheek and skin irritations (24–26), mucosal irritation (4, 24, 27), inflammation of nasal tissues (4, 24), vomiting (28), infections (24), bleeding (24) and feeding difficulties (26). Our present study aligns with previous findings, with a majority of centers reporting instances of mucosal and skin irritation. Importantly, it should be noted that while these side effects are common, they do not typically impact parental or caretaker compliance with the treatment regimen

### Recall visits

One of the primary challenges associated with Nasoalveolar Molding (NAM) treatment is the frequency of recall visits, which places a significant burden on caretakers. Studies indicate that approximately 10–15 appointments are typically required during the course of treatment (29, 30). There is notable variation in the frequency of these recall intervals across different studies, with the majority advocating for weekly reviews (6, 30, 31), while others suggest biweekly intervals (32). Interestingly, findings from our study reveal an equal preference among treatment centers for both weekly and biweekly recall schedules.

### NAM technique

With the increasing adoption of Nasoalveolar Molding (NAM) treatment over the past two decades, various techniques and modifications have emerged worldwide (4, 33–36). Among these, the Grayson and Figueroa techniques are the most widely employed. Several studies, albeit of varying quality, have compared these techniques, indicating comparable success rates with differences primarily observed in appointment frequency and side effects (31, 34, 37, 38). In our present study, more than half of respondents reported using the Grayson technique, while 15% employed the Figueroa technique and small percentage used Liou's technique.

## Limitation of the study

The survey was conducted across a limited number of comprehensive cleft centers, which may not fully represent the entire landscape of cleft care in India. Furthermore, while the participating centers included a mix of public and private (NGO-funded) facilities, the majority (64%, *n* = 29) were NGO-funded. It is noteworthy that in India, 86% of cleft centers are operated by private NGOs (14). A second limitation of this survey is its focus solely on healthcare providers and did not gather perspectives from caregivers.

## Conclusions

1. The survey reveals that 85% of comprehensive cleft centers in India offer NAM treatment, although nearly half of these centers provide it routinely for fewer than 20% of their patients.
2. There is a consensus that NAM is beneficial for both unilateral and bilateral clefts and that orthodontists should administer the treatment. The Grayson's method is the most commonly used technique, with an equal number of centers recalling patients at one-week and two-week intervals.
3. While there is general agreement on the effectiveness of NAM, 15% of participants were unsure about its efficacy. Only about one-fifth of respondents found the NAM treatment challenging.
4. The most frequently reported side effects were cheek and mucosal irritation. Despite these issues, all respondents would recommend NAM treatment to their family and friends.

## Data Availability

The original contributions presented in the study are included in the article/Supplementary Material, further inquiries can be directed to the corresponding authors.
